# Supplementary figures and images for: The Group B Streptococcal surface antigen I/II protein, BspC, interacts with host vimentin to promote adherence to brain endothelium and inflammation during the pathogenesis of meningitis
Source: PLoS Pathog. 2019 Jun 10;15(6):e1007848. doi: 10.1371/journal.ppat.1007848 (PMC6586375; doi:10.1371/journal.ppat.1007848)

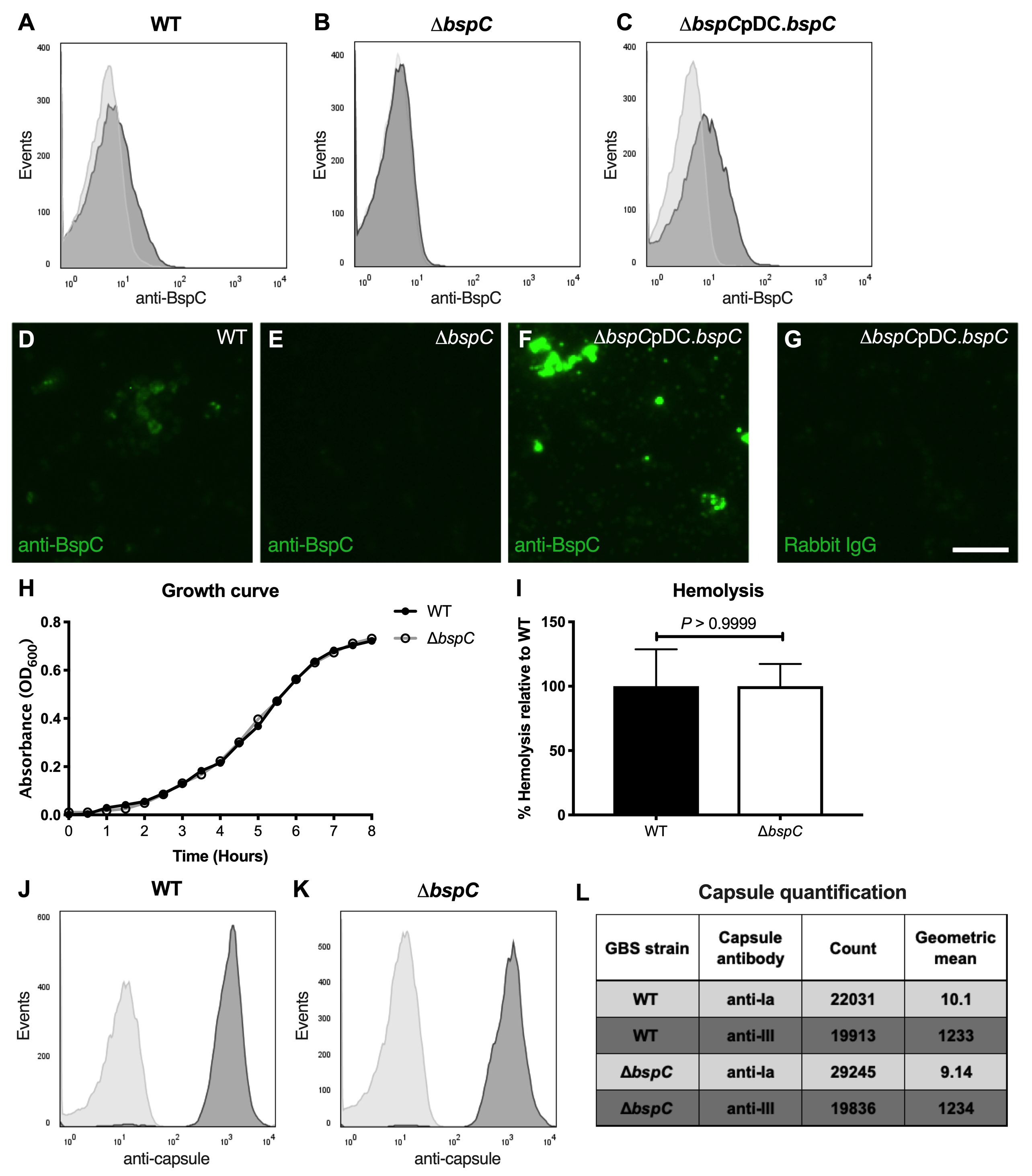

Supplement: S1 Fig — (A-C) Flow cytometry using a polyclonal rabbit antibody to BspC to show expression of BspC in WT COH1 (A), ΔbspC mutant (B), and the complemented (C) GBS strains. (D-G) Immunofluorescent staining of WT COH1 (D), ΔbspC mutant (E), and the complemented (F) GBS strains using the BspC antibody to show surface localization of BspC protein. (G) Negative staining control. Scale bar is 5 μm. (H) Growth curves for WT GBS and the ΔbspC mutant in THB. (I) Hemolysis assay comparing hemolysis of sheep blood by WT GBS and the ΔbspC mutant. Representative data of one of at least three independent experiments are shown. Error bars represent the standard deviation of mean in one experiment. Data were analyzed using an unpaired t test. (J and K) Flow cytometry using a monoclonal antibody to the serotype III capsule to determine the presence of capsule in WT GBS (J) and the ΔbspC mutant (K) and a monoclonal antibody to the serotype Ia capsule as an isotype control. (L) Quantification of capsule flow cytometry data shown in (J) and (K). (TIFF) [file ppat.1007848.s001.tiff]

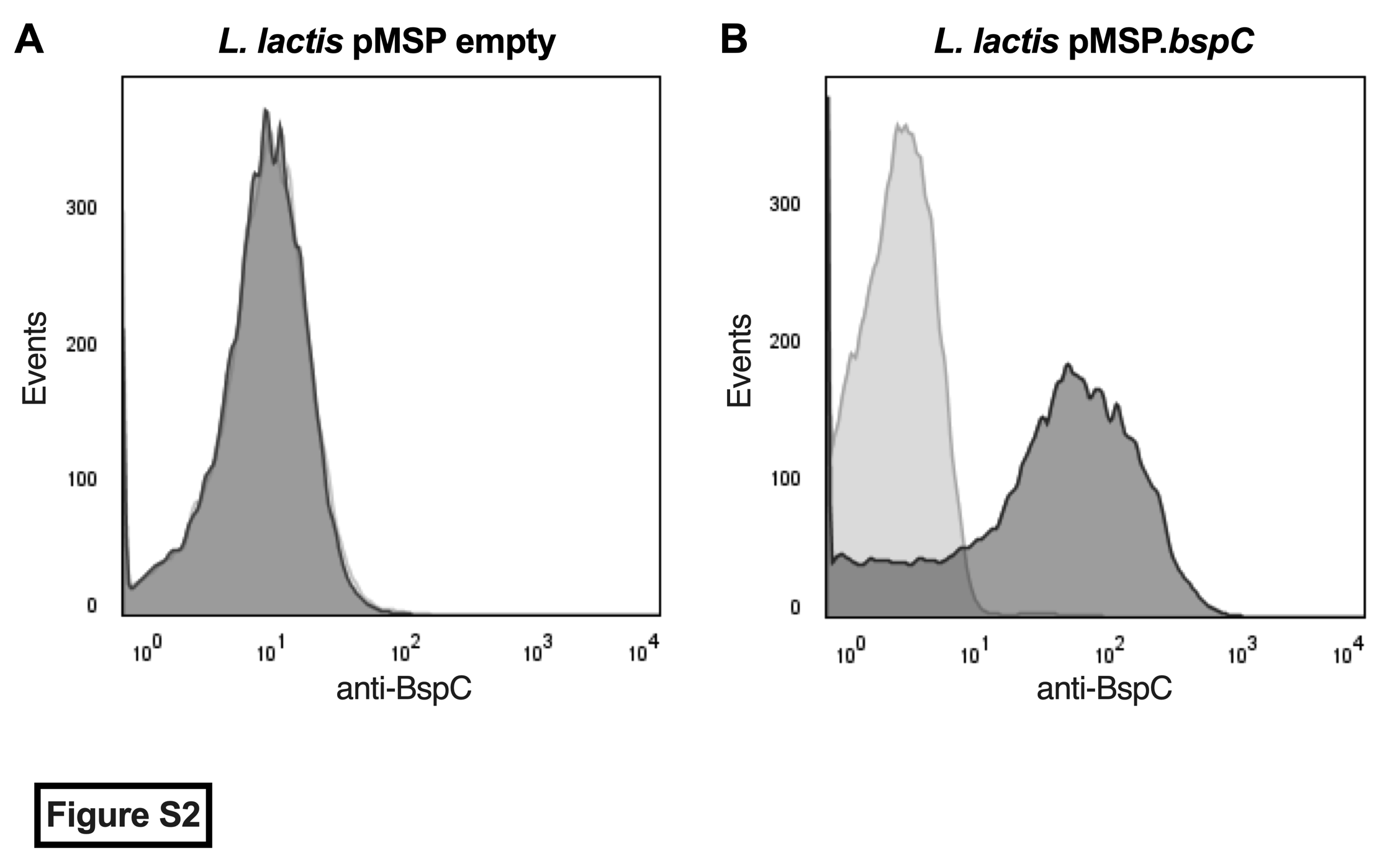

Supplement: S2 Fig — Flow cytometry to show BspC surface expression in L. lactis containing the pMSP empty plasmid (A) and L. lactis containing the pMSP.bspC vector (B). (TIFF) [file ppat.1007848.s002.tiff]

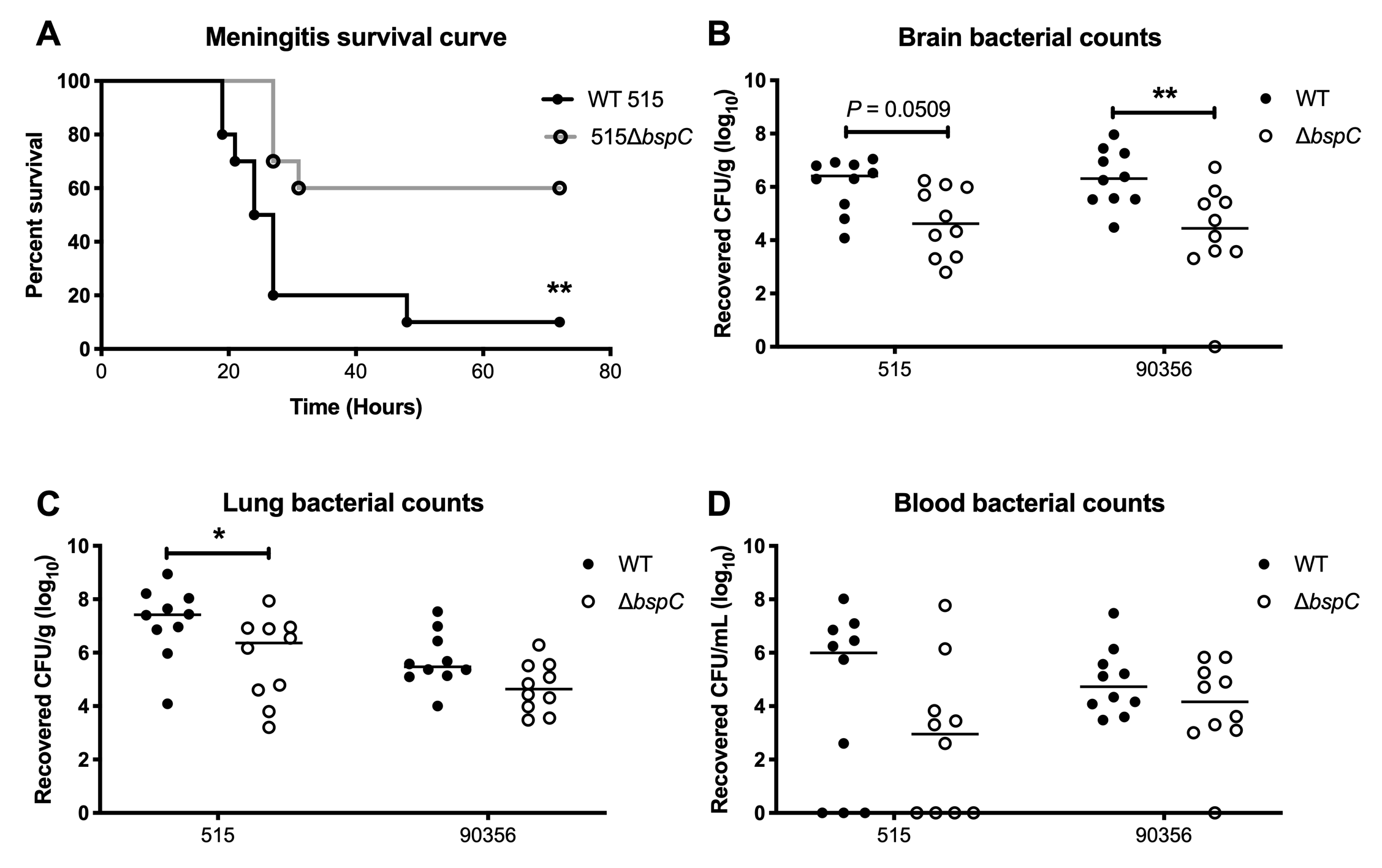

Supplement: S3 Fig — (A) Kaplan-Meier plot showing survival of mice challenged with either WT 515 GBS or the isogenic ΔbspC mutant. (B-D) Tissue bacterial counts for mice infected with WT 515 and 90356 GBS and the isogenic ΔbspC mutants. 48h post-infection, mice were sacrificed and bacterial loads in brain (B), lung (C), and blood (D) were quantified. Statistical analysis: (A) Log-rank test. (B-D) Two-way ANOVA with Sidak’s multiple comparisons test. *, P < 0.0005; **, P < 0.005. (TIFF) [file ppat.1007848.s003.tiff]

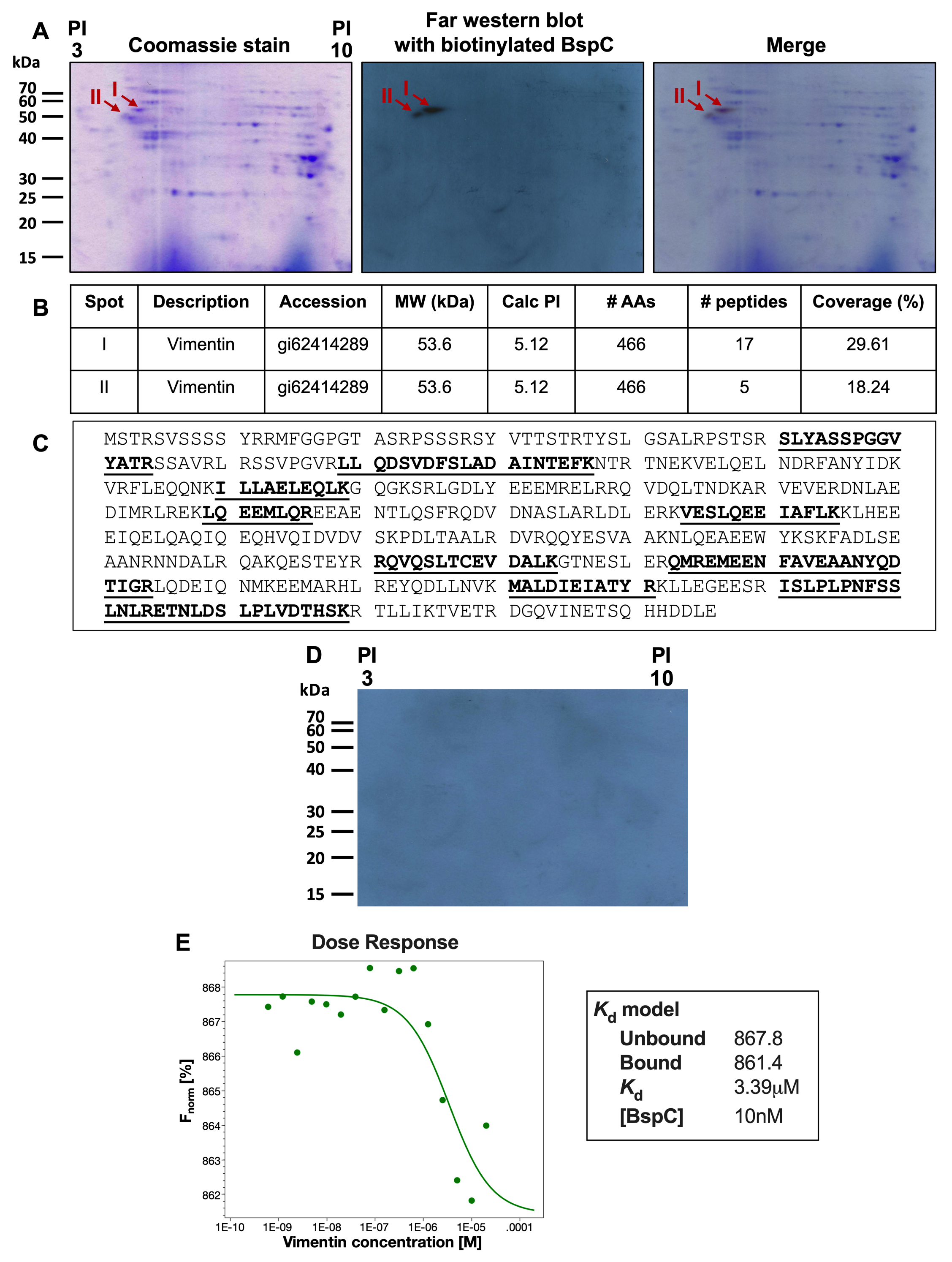

Supplement: S4 Fig — (A) Far western blot analysis of hBMEC membrane proteins using biotinylated BspC protein. Two spots (I and II) were identified on the x-ray film and aligned to the Coomassie stained gel. (B) Electrospray ionization-tandem mass spectrometry identifies spots I and II as vimentin. (C) The amino acid sequence of human vimentin, with the peptide sequences identified in the MS analysis underscored and bolded. (D) Control Far Western blot with the streptavidin antibody conjugated to HRP only. (E) Representative MST dose response curve quantifying the dissociation constant for the interaction between BspC and vimentin. (TIFF) [file ppat.1007848.s004.tiff]

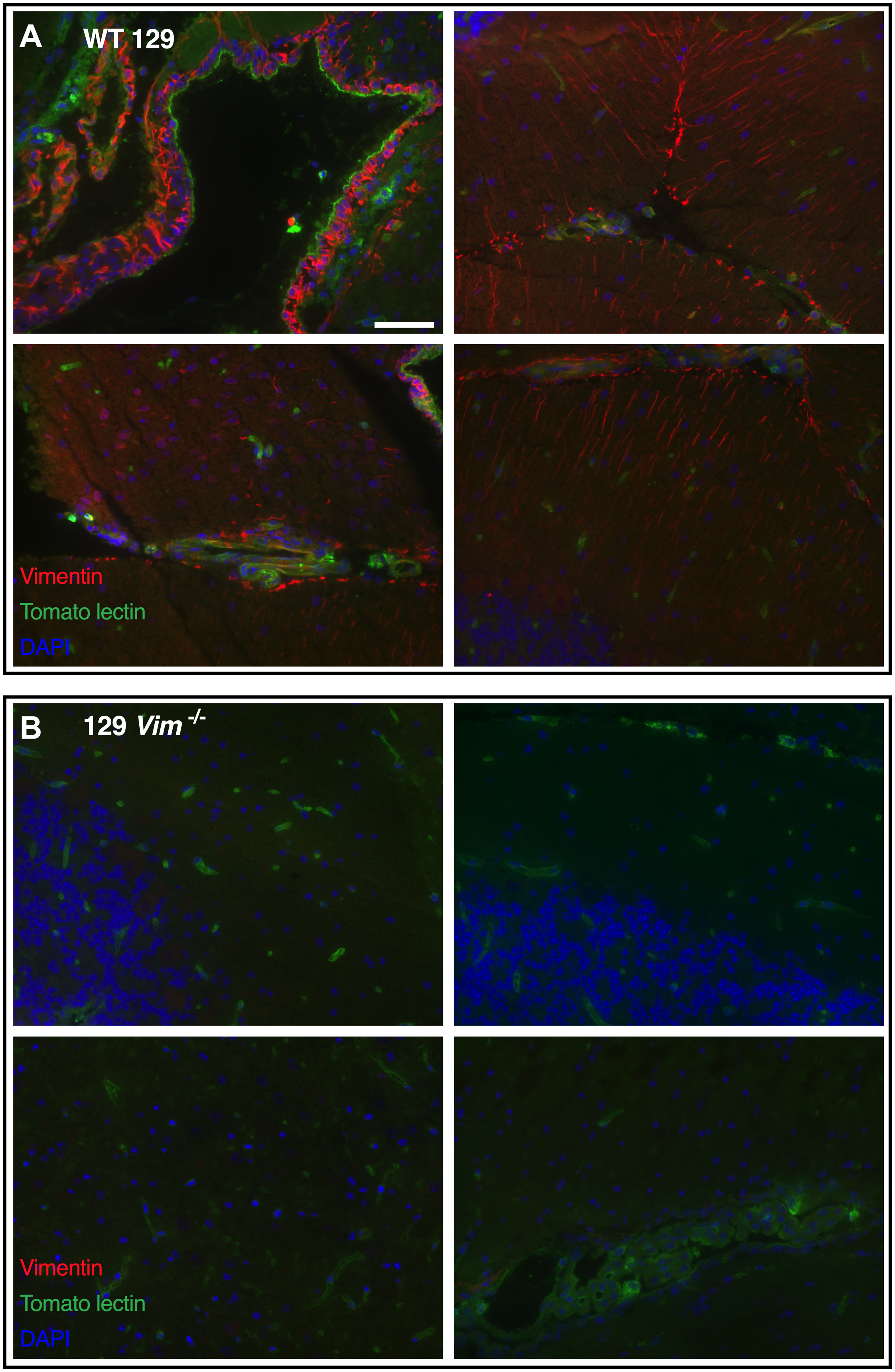

Supplement: S5 Fig — Immunofluorescent staining of WT 129 (A) and 129 Vim-/- (B) brain tissue sections with an antibody to vimentin and with tomato lectin to label blood vessels. Nuclei were labelled with DAPI. Scale bar is 50 μm. (TIFF) [file ppat.1007848.s005.tiff]

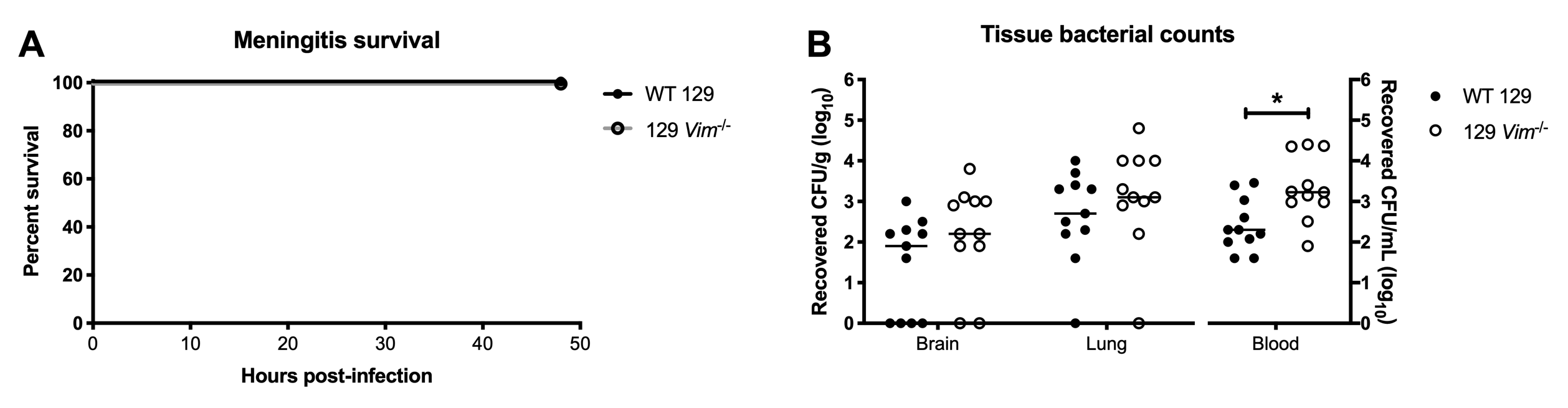

Supplement: S6 Fig — (A) Kaplan-Meier plot showing survival of WT 129 mice or 129 Vim-/- mice challenged with GBS ΔbspC mutant. (B) 48h post-infection, mice were sacrificed and bacterial loads in brain, lung, and blood were quantified. (TIFF) [file ppat.1007848.s006.tiff]
